# Risk Factors for Helminth, Malaria, and HIV Infection in Pregnancy in Entebbe, Uganda

**DOI:** 10.1371/journal.pntd.0000473

**Published:** 2009-06-30

**Authors:** Patrick William Woodburn, Lawrence Muhangi, Stephen Hillier, Juliet Ndibazza, Proscovia Bazanya Namujju, Moses Kizza, Christine Ameke, Nicolas Emojong Omoding, Mark Booth, Alison Mary Elliott

**Affiliations:** 1 MRC/UVRI Uganda Research Unit on AIDS, Entebbe, Uganda; 2 University of Birmingham, Birmingham, United Kingdom; 3 University of Durham, Durham, United Kingdom; 4 London School of Hygiene and Tropical Medicine, London, United Kingdom; London School of Hygiene & Tropical Medicine, United Kingdom

## Abstract

**Background:**

Infections during pregnancy may have serious consequences for both mother and baby. Assessment of risk factors for infections informs planning of interventions and analysis of the impact of infections on health outcomes.

**Objectives:**

To describe risk factors for helminths, malaria and HIV in pregnant Ugandan women before intervention in a trial of de-worming in pregnancy.

**Methods:**

The trial recruited 2,507 pregnant women between April 2003 and November 2005. Participants were interviewed and blood and stool samples obtained; location of residence at enrolment was mapped. Demographic, socioeconomic, behavioral and other risk factors were modelled using logistic regression.

**Results:**

There was a high prevalence of helminth, malaria and HIV infection, as previously reported. All helminths and malaria parasitemia were more common in younger women, and education was protective against every infection. Place of birth and/or tribe affected all helminths in a pattern consistent with the geographical distribution of helminth infections in Uganda. Four different geohelminths (hookworm, *Trichuris*, *Ascaris* and *Trichostrongylus*) showed a downwards trend in prevalence during the enrolment period. There was a negative association between hookworm and HIV, and between hookworm and low CD4 count among HIV-positive women. Locally, high prevalence of schistosomiasis and HIV occurred in lakeshore communities.

**Conclusions:**

Interventions for helminths, malaria and HIV need to target young women both in and out of school. Antenatal interventions for malaria and HIV infection must continue to be promoted. Women originating from a high risk area for a helminth infection remain at high risk after migration to a lower-risk area, and vice versa, but overall, geohelminths seem to be becoming less common in this population. High risk populations, such as fishing communities, require directed effort against schistosomiasis and HIV infection.

## Author Summary

Infections in pregnancy can cause miscarriage, stillbirth, maternal mortality, and low birth weight and have other long-term complications for mother and baby, although the full impact of many infections, particularly worm infections, is not yet fully understood. There is a high burden of infectious disease in many developing countries. In this analysis, we identified which factors put pregnant women in Entebbe, Uganda, at particular risk for worm infections, malaria, HIV, and, where possible, rarer infections including syphilis. The women in this study, and their children, will be followed up to determine the long-term effects of exposure of the fetus to these maternal infections on health during childhood. The findings of this baseline analysis will help in the interpretation of the long-term outcomes. The findings also highlight which groups are most at risk of each infection, and this may help in targeting interventions to prevent, treat, or mitigate the impact of infections in pregnancy.

## Introduction

Approximately one third of the world's population is infected with helminths and, in recent years, helminth control has been the focus of renewed interest on account of the concomitant direct disease burden [1],[2]. At the same time, researchers have become more interested in the effects helminths have on immunoregulation, and the possible consequences this may have for susceptibility to other infections, for vaccine efficacy, and for immunologically mediated conditions such as allergy and autoimmune disease [3]. Immunological effects may vary between different helminth species, between low and high intensity of infection, between chronic and acute infections, or between mono- and poly-parasitic infections, and may also differ in unique populations, such as pregnant women and very young children. Risk factors for helminth infection depend on the route of transmission and lifecycle of the helminth, but are usually related to hygiene, sanitation and, for some species, environmental conditions required for the intermediate hosts or for a free-living soil-dwelling stage. The degree to which immunity may be developed to helminths depends very much on the species, leading to varied age distributions, but long-term chronic infection over several years has been observed for many species [4]. The varied environmental conditions required lead to varied geographic distributions of helminths both within and between countries and regions, and influenced by climate factors and proximity to water bodies [4],[5]. The primary recommended public health intervention for control of soil transmitted helminths and schistosomes is regular mass treatment, particularly of high-risk groups, backed up by improved access to clean water, sanitation and health education [6]. Pregnancy itself may increase susceptibility to helminths, but this is uncertain; a recent study from Gabon showed an increased prevalence in pregnancy [7], but another from Thailand found no association [8].

*Plasmodium falciparum*, HIV and syphilis infection are known to be major health concerns in pregnancy [9]–[14]. Pregnant women are particularly vulnerable to *P. falciparum* infection, especially in the first pregnancy [9], and protective interventions against malaria include intermittent presumptive treatment and the use of bed nets [15],[16]. HIV-positive women tend to experience faster CD4 decline during and after pregnancy [17], with risk factors for HIV and syphilis including risky sexual behavior and multiple or unstable partnerships [18].

The Entebbe Mother and Baby Study (EMABS; trial registration number ISRCTN3289447) was designed to examine the effect of routine de-worming with albendazole and praziquantel in pregnancy and early childhood [19]. This analysis describes the prevalence of and risk factors for helminth infections, malaria, HIV and syphilis in pregnant women enrolled into EMABS at baseline. The results have potential for application in the development of strategies for prevention and control of infections during pregnancy, and, within the study, to identify possible confounders for future analyses.

## Methods

### Study population and procedures

Participants were enrolled from women attending the antenatal clinic at Entebbe General Hospital between April 2003 and November 2005, as previously described [19],[20]. Briefly, after giving written informed consent, women in the second or third trimester of pregnancy were enrolled if: they lived within Entebbe or the neighbouring subcounty, Katabi; planned to deliver at the hospital; were willing to participate and were willing to take an HIV test. To avoid neglect of symptomatic helminth infections, women were excluded if they were anaemic or had clinical evidence of severe liver disease, or history of diarrhoea with blood in stool. They were also excluded if they had a history of adverse reaction to anthelmintic drugs; if the pregnancy was abnormal; or if they had enrolled previously in an earlier pregnancy. At enrolment they were interviewed for demographic and socioeconomic information, besides proximate risk factors for infections, and provided a blood and a stool sample before being treated with the study intervention.

### Diagnosis and treatment of infections

Intestinal helminths were identified by the Kato-Katz method [21],[22]. Two Kato-Katz slides were prepared from each stool sample, then examined within 30 minutes for hookworm and again the following day for ova of other parasites. *Strongyloides* was diagnosed by charcoal culture [23]. *Mansonella perstans* was identified from the blood sample by a modified Knott's method [24].

HIV was identified using a triple rapid test serial testing algorithm [20]. *P. falciparum* parasitemia was identified by examination of thick blood films. Women were screened for syphilis on a Rapid Plasma Reagin (RPR) test, with positive results being further tested for active syphilis, based on a definition of RPR titer> = 1∶4 and a positive *Treponema Pallidum* Haemagglutination Assay (TPHA) result (both kits supplied by BIOTEC Laboratories Ltd., UK).

Helminth infections were treated according to study protocol, either during pregnancy (the trial intervention) or after delivery. In accordance with recommendations from the Ugandan Ministry of Health: all women received intermittent presumptive treatment for malaria with sulphadoxine-pyrimethamine; HIV-positive women received nevirapine for prevention of mother-to-child HIV transmission (PMTCT), and, if they had a low CD4-count, were referred for treatment; RPR-positive women received benzathine penicillin.

### Statistical methods

**Figure 1:**
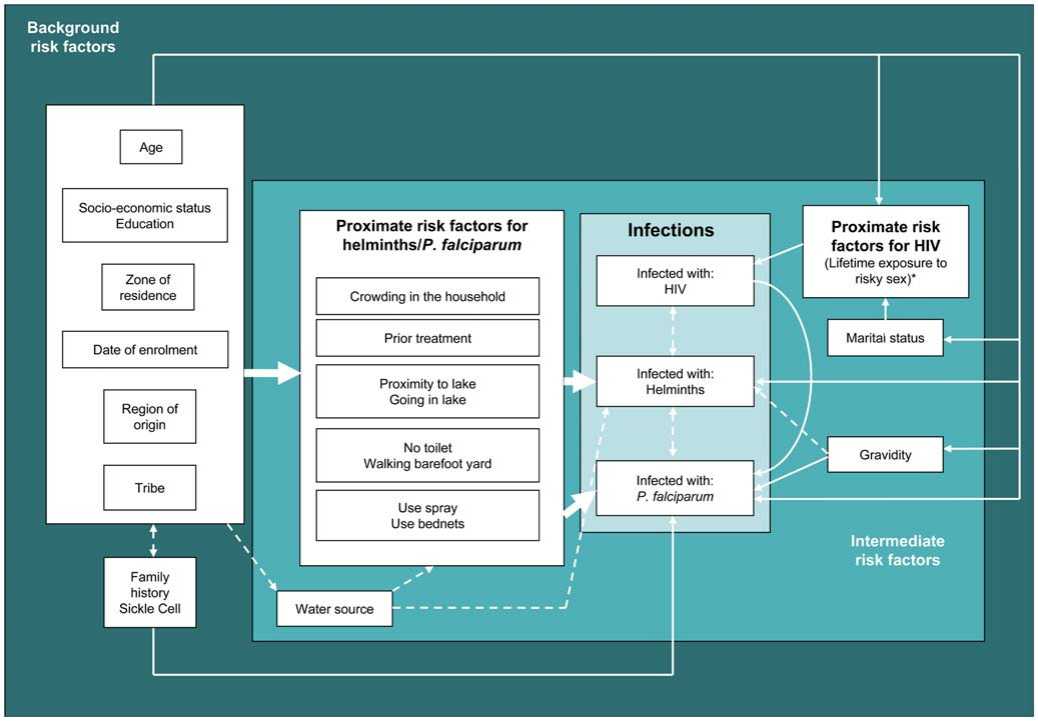
Causal diagram showing hypothetical hierarchy of risk factors for helminth, malaria and HIV infection. Expected associations are shown using solid lines and arrows; possible associations are shown using dotted lines and arrows. * Proximate risk factors considered for HIV infection included exposure to a partner known or likely to have HIV, any perceived exposure to HIV, history of sexually transmitted infections, alcohol use or high risk occupation.

Analyses were performed using Stata SE 9.2. We drew up a causal diagram of potential risk factors for each infection (Figure 1), and then considered the crude effect of each risk factor of interest, to develop a list of infection-specific background, intermediate and proximate risk factors. Background risk factors were adjusted only for each other, intermediate for background and intermediate, and proximate for all other risk factors.

Background risk factors were mostly the same for all infections. Maternal age, education and tribe were considered. Maternal place of birth was defined based on district of birth and categorized by Uganda Census regional definitions [25], plus two additional categories of Outside Uganda and Wakiso (the study district, a component of the Central region). Socioeconomic variables were considered using a principal component approach which allowed three dominant variables to be identified. These comprised building materials, number of rooms and items owned. Responses to these variables were used to develop a score with six levels for household socioeconomic status (SES) [20]. Zone of residence was based on geographical positioning system (GPS) data [26], using a simplified version of the zones in Hillier et al [26] which divided the area into the town of Entebbe, the adjacent trading centres of Abaita and Nkumba, the fishing village of Kigungu, the roadside villages of Katabi and rural areas of Katabi extending south to Lake Victoria. For *P. falciparum*, we also considered a family history of sickle cell disease.

Intermediate risk factors considered were: for helminths, HIV status, gravidity and type of water source; for *P. falciparum*, HIV status, gravidity and helminth infections; and for HIV, marital status.

Proximate risk factors considered for helminths were prior anthelmintic treatment, crowding in the household, exposure to Lake Victoria through swimming or bathing, home toilet facilities and walking barefoot; for *P. falciparum*, use of bed nets, insecticide sprays, and antimalarial treatment. Proximate risk factors considered for HIV were those likely to be strongly associated with lifetime exposure to risky sexual activity: reported exposure to a partner known or likely to have HIV, any perceived exposure to HIV, history of sexually transmitted infections, alcohol use or high risk occupation; and gravidity; we also considered a composite variable for an effect modification of perceived exposure on partner exposures.

Having identified potential risk factors of interest we obtained adjusted odds ratios using logistic regression. For each infection a final model was developed which reconsidered the significance of each risk factor by stepwise elimination, at a p-value of <0.1 for inclusion in the model. Where the risk factor could be considered either as a continuous or a categorical variable, we compared the fit of the two versions individually using a likelihood ratio test, with the simpler model as the null hypothesis.

In assessing coinfections, we did not consider helminths as risk factors for other helminths - this is the subject of a separate analysis. However, we did consider HIV as a risk factor for all helminths and for *P. falciparum*, and helminths as risk factors for *P. falciparum*.

### Ethics statement

Approval was given for all work described by the Science and Ethics Committee, Uganda Virus Research Institute; the Uganda National Council for Science and Technology; and the London School of Hygiene and Tropical Medicine. Written informed consent was given by all pregnant women at enrolment.

## Results

### Demographic characteristics

As reported elsewhere, we assessed 11,783 women for inclusion of whom 3163 were considered eligible and screened; 2515 women were enrolled of whom eight were later excluded because of enrolment during an earlier pregnancy, leaving 2507 for analysis [20]. The principal reasons for ineligibility and exclusion have been reported but, of note, only 15 were ineligible because of diarrhea with blood in the stool and 17 excluded for anaemia; none was identified as having clinical evidence of liver disease.

Among the 2507 women included, reported ages ranged from 14 to 47 years, median 23. The majority (54%) had primary education only or no formal education at all; 63% described themselves as unemployed or housewives and 82% had a personal income below 30,000 Ugandan shillings (∼US$20) per month. Eighty-four percent of women were married, and the most common occupational groups of the male partner were professional (36%) and unskilled manual (31%). Nineteen percent of study women were born in the study district of Wakiso, 43% elsewhere in central Uganda, 16% in west Uganda, 8% in north Uganda, 13% in east Uganda and 2% outside Uganda; 49% were from the local Baganda tribe with less than 10% from any other individual tribe. The number of prior pregnancies for each woman ranged from zero to 10, median 2. Forty-six percent were enrolled in their second trimester, 54% in the third.

Among 648 women screened but not enrolled median age was lower (21 years, p<0.001) and a higher proportion were primigravida (33% compared to 28%, p = 0.001). There were no differences in education, occupation, income or marital status, and a similar proportion (50%) were Baganda, although numbers varied in other tribal groups.

### Infection prevalence

Hookworm infection was detected in 1112 of 2498 women (44.5%), *M. perstans* in 531 of 2499 (21.2%), *S. mansoni* 458 of 2498 (18.3%), *Strongyloides* 306 of 2485 (12.3%), *Trichuris* 226 of 2498 (9.0%), and *Ascaris* 58 of 2498 (2.3%). Of the 2477 tested for all helminth infections, 1693 (68.3%) had at least one helminth infection (defined as any of the above, *Trichostrongylus* (26 cases), *Hymenolypsis nana* (4 cases), *Enterobius vermicularis* (1 case) or *Loa Loa* (1 case)). *P. falciparum* was detected in 268 of 2459 women (10.9%) (*P. vivax*, *ovale* or *malariae* were not detected), HIV in 299 of 2507 (11.9%) and active syphilis in 18 of 2507 (1.1%) (110 women had a positive RPR test).

Among the 648 women screened but not enrolled the prevalence of malaria was similar (13.0%). The prevalence of HIV infection was significantly higher (18.5%; p<0.001) but the median CD4 counts were similar between HIV-positive women who were enrolled or screened but not enrolled (449 and 457 cells/µl, respectively, p = 0.40). The prevalence of *M. perstans was similar* (23.9%) but the prevalence of other helminths could not be assessed as many of these women failed to return with a stool sample.

### Treatment history

Anthelmintics had ever been taken previously by 1121 women (45%); 227 women (9%) had taken them in this pregnancy, of whom 127 had taken mebendazole, 41 albendazole, 14 levamisole, five praziquantel and 53 an unknown drug for worms. Of these 227 women, 215 had taken only one type, 11 two types and one woman three types of anthelmintic. Antimalarials had been taken earlier in the pregnancy by 2391 women (96%), of whom 2335 reported taking sulphadoxine-pyrimethamine, 628 chloroquine, 155 quinine, 117 co-trimoxazole, nine sulphalene-pyrimethamine, and three an unknown antimalarial; 1613 women took one type of antimalarial only, 701 two, 76 three and one woman four. Six of 299 HIV-positive women were on antiretroviral therapy.

### Associations with helminth infections

Crude and adjusted odds ratios for factors associated in the final model with hookworm, *M. perstans*, *S. mansoni*, *Strongyloides* and *Trichuris* are shown in Tables 1, 2, S1, S2 and S3. Young age and lack of education were risk factors for all species. Higher household socioeconomic status showed a protective association with hookworm, *M. perstans* and *Strongyloides* infections, but no association with *S. mansoni* or *Trichuris*. Tribe and region of origin each showed associations with particular helminth infections, both before and after adjusting for mutual confounding. For example, a woman had higher odds of *M. perstans* infection if she was a Musoga or born in Central Uganda, but lower odds if born in the North, whereas women born in the North or in Wakiso had higher odds of *S. mansoni* infection. Hookworm, *S. mansoni* and *Trichuris* showed variability in prevalence within the study area, as reported elsewhere [26]. Later date of enrolment showed a continuous protective association for hookworm and *Trichuris*, but no association for *S. mansoni* or *Strongyloides*. HIV infection showed a negative association with hookworm, but no association with other helminths. Among HIV-positive mothers, those with a lower CD4 were less likely than those with a higher CD4 count to have hookworm infection: hookworm prevalence ranged from 10% among women with a CD4 below 100 to 46% among women with a CD4 of 500 or above, similar to the prevalence of 45% among women without HIV; the odds ratio for hookworm infection per 100 cells/µl increase in CD4 count, (adjusted for age, education, tribe, place of birth, household socioeconomic status (SES), zone of residence and date enrolled) was 1.16 (95% confidence interval 1.02–1.32). Gravidity showed a crude association with hookworm, *M. perstans* and *Trichuris*, but no association after adjusting for age and education. Walking around the yard barefoot showed only a weak crude association with hookworm and *Strongyloides*, and no evidence of an effect after adjusting for confounders. Having a pit latrine or toilet versus no toilet showed a crude protective effect against some hygiene-related helminth infections (hookworm and *Trichuris*), but no effect after adjusting for socioeconomic status variables. The main water source used showed a crude association with the lake-derived *S. mansoni* and with the hygiene-related *Strongyloides* and *Trichuris*, although only the *S. mansoni* association was retained in the adjusted model. Swimming/bathing in the lake, however, showed crude and adjusted associations with all three of these infections. Distance to the lake showed a crude association with *S. mansoni*, *Strongyloides* and *Trichuris*, but no association after adjusting for zone of residence. Prior anthelmintics showed a strong protective association after adjustment on hookworm, *M. perstans* and *Strongyloides*, but only an unadjusted effect on *S. mansoni* and no effect on *Trichuris*. Having a room to herself showed a protective effect on hookworm infection, but otherwise household crowding showed no effect on helminths.

**Table 1:** Hookworm.

Level	Risk Factor	Crude OR	Adjusted OR	(95% CI)	LR p-value
Background^1^	Age (continuous, per year)	0.94	0.94	(0.93–0.96)	<0.0001
	Education (continuous, per stage)	0.63	0.65	(0.57–0.73)	<0.0001
	Tribe				0.03
	Muganda	1.0	1.0		
	Munyankole	1.11	1.54	(1.03–2.32)	
	Mutoro	0.66	0.91	(0.53–1.57)	
	Musoga	1.39	1.83	(1.05–3.19)	
	Luo	0.69	1.27	(0.69–2.33)	
	Munyarwanda	1.22	0.99	(0.68–1.44)	
	Other	0.70	0.96	(0.69–1.32)	
	Place of birth				0.0009
	Wakiso district	1.0	1.0		
	Other central region district	1.38	1.34	(1.06–1.70)	
	Western region	0.90	0.79	(0.53–1.18)	
	Northern region	0.66	0.59	(0.34–1.04)	
	Eastern region	1.08	0.99	(0.64–1.53)	
	Outside Uganda	0.54	0.43	(0.20–0.98)	
	Household SES group (continuous, per unit)	0.78	0.84	(0.78–0.90)	<0.0001
	Zone of residence				<0.0001
	Entebbe	1.0	1.0		
	Kigungu	2.09	1.91	(1.43–2.55)	
	Abaita/Nkumba	1.01	1.00	(0.81–1.24)	
	Katabi, near main road	1.04	1.21	(0.90–1.63)	
	Katabi, away from main road	2.23	2.07	(1.49–2.87)	
	Unmapped				
	Date enrolled (continuous, per year)	0.83	0.76	(0.67–0.86)	<0.0001
Intermediate^2^	HIV positive	0.77	0.72	(0.55–0.94)	0.02
	CD4 count (continuous, per 100 cell increase, HIV+ only)	1.20	1.16	(1.02–1.32)	0.02
	*Water source*				
	*Tap*	*1.0*	*1.0*		*0.5*
	*Stand Pipe*	*1.17*	*1.00*	*(0.82–1.22)*	
	*Bore Hole*	*1.48*	*1.13*	*(0.78–1.62)*	
	*Well*	*0.89*	*0.73*	*(0.50–1.08)*	
	*Lake*	*1.86*	*0.93*	*(0.59–1.47)*	
	*Primigravida*	*1.19*	*0.91*	*(0.73–1.14)*	*0.4*
Proximate^3^	Any prior anthelmintic treatment				<0.0001
	Never	1.0	1.0		
	Only prior to this pregnancy	0.44	0.51	(0.42–0.61)	
	During this pregnancy	0.23	0.27	(0.19–0.39)	
	Own room	0.58	0.47	(0.29–0.77)	0.002
	*Ever swims/bathes in lake*	*0.83*	*0.89*	*(0.74–1.07)*	*0.2*
	*Home toilet facilities*				*0.9*
	*Pit latrine*	*1.0*	*1.0*		
	*Flush toilet*	*0.74*	*1.10*	*(0.77–1.58)*	
	*None*	*1.76*	*0.94*	*(0.45–1.98)*	
	*Ever walks in yard barefoot*	*1.26*	*1.15*	*(0.93–1.43)*	*0.2*

1, 2 Background and intermediate risk factors adjusted for age, education, tribe, place of birth, household socioeconomic status (SES), zone of residence and date enrolled.

3 Proximate risk factors adjusted for age, education, tribe, place of birth, household socioeconomic status (SES), zone of residence, date enrolled, HIV status, prior anthelmintic treatment and own room.

Variables that were omitted from the final models are shown in italics.

**Table 2:** *Schistosoma mansoni*.

Level	Risk Factor	Crude OR	Adjusted OR	(95% CI)	LR p-value
Background^1^	Age (continuous, per year)	0.98	0.98	(0.96–1.00)	0.02
	Education (continuous, per stage)	0.73	0.77	(0.66–0.90)	0.001
	Place of birth				<0.0001
	Wakiso district	1.0	1.0		
	Other central region district	0.32	0.30	(0.23–0.39)	
	Western region	0.18	0.17	(0.11–0.27)	
	Northern region	1.41	1.44	(1.01–2.06)	
	Eastern region	0.32	0.34	(0.23–0.49)	
	Outside Uganda	1.19	1.07	(0.53–2.15)	
	Zone of residence				<0.0001
	Entebbe	1.0	1.0		
	Kigungu	1.86	1.60	(1.12–2.27)	
	Abaita/Nkumba	1.67	1.49	(1.15–1.95)	
	Katabi, near main road	0.90	0.86	(0.57–1.30)	
	Katabi, away from main road	2.28	2.43	(1.68–3.52)	
	Unmapped	1.36	1.45	(0.66–3.16)	
	*Tribe*				*0.3*
	*Muganda*	*1.0*	*1.0*		
	*Munyankole*	*0.45*	*1.02*	*(0.57–1.84)*	
	*Mutoro*	*0.73*	*1.83*	*(0.88–3.81)*	
	*Musoga*	*1.00*	*2.08*	*(1.02–4.23)*	
	*Luo*	*2.90*	*1.72*	*(0.91–3.27)*	
	*Munyarwanda*	*0.88*	*1.02*	*(0.62–1.68)*	
	*Other*	*1.11*	*1.35*	*(0.91–2.00)*	
	*Household SES group (continuous, per unit)*	*0.96*	*1.03*	*(0.94–1.13)*	*0.5*
	*Date enrolled (continuous, per year)*	*0.95*	*0.96*	*(0.82–1.12)*	*0.6*
Intermediate^2^	Water source				
	Tap				0.04
	Stand Pipe	1.0	1.0		
	Bore Hole	1.08	1.15	(0.89–1.48)	
	Well	1.05	0.80	(0.49–1.30)	
	Lake	1.43	1.28	(0.81–2.01)	
	*HIV positive*	*1.06*	*1.08*	*(0.78–1.51)*	*0.2*
	*Primigravida*	*0.97*	*0.97*	*(0.73–1.29)*	*0.9*
Proximate^3^	Ever swims/bathes in lake	2.61	2.39	(1.85–3.09)	<0.0001
	*Any prior anthelmintic treatment*				*0.4*
	*Never*	*1.0*	*1.0*		
	*Only prior to this pregnancy*	*0.97*	*1.03*	*(0.81–1.31)*	
	*During this pregnancy*	*0.50*	*0.76*	*(0.47–1.22)*	
	*Own room*	*1.16*	*1.05*	*(0.62–1.80)*	*0.8*

1, 2 Background and intermediate risk factors adjusted for age, education, place of birth, and zone of residence.

3 Proximate risk factors adjusted for age, education, place of birth, zone of residence and water source.

Variables that were omitted from the final models are shown in italics.

### Associations with malaria parasitemia

Factors associated with *P. falciparum* parasitemia are shown in Table 3. Age and education both showed a strong protective effect. Zone of residence showed good evidence of an association, as described elsewhere [26]. Tribe showed some evidence of a crude association with malaria, but no effect was seen in the adjusted model. Place of birth and household SES showed no association, and there was no evidence of a change over time other than seasonal effects (data not shown). HIV infection increased the risk of having malaria, while using a net, using treatment, and being multigravid were all protective. No effect was seen of hand spraying against mosquitoes, or of a reported family history of sickle cell disease. Both hookworm and *M. perstans* showed an association with *P. falciparum* as described elsewhere [26].

**Table 3:** *P. falciparum* parasitemia.

Level	Risk Factor	Crude OR	Adjusted OR	(95% CI)	LR p-value
Background^1^	Age (continuous, per year)	0.90	0.90	(0.87–0.93)	<0.0001
	Education (continuous, per stage)	0.81	0.79	(0.65–0.96)	0.02
	Zone of residence				0.0001
	Entebbe	1.0	1.0		
	Kigungu	0.33	0.30	(0.15–0.58)	
	Abaita/Nkumba	1.09	1.03	(0.75–1.41)	
	Katabi, near main road	1.23	1.21	(0.79–1.83)	
	Katabi, away from main road	1.65	1.56	(1.02–2.39)	
	Unmapped	0.95	0.74	(0.29–1.93)	
	*Tribe*				*0.1*
	*Muganda*	*1.0*	*1.0*		
	*Munyankole*	*0.60*	*0.61*	*(0.37–1.02)*	
	*Mutoro*	*0.75*	*0.72*	*(0.36–1.43)*	
	*Musoga*	*0.87*	*0.94*	*(0.50–1.77)*	
	*Luo*	*0.41*	*0.47*	*(0.22–1.00)*	
	*Munyarwanda*	*0.57*	*0.54*	*(0.28–1.04)*	
	*Other*	*0.74*	*0.85*	*(0.60–1.19)*	
	*Place of birth*				*0.8*
	*Wakiso district*	*1.0*	*1.0*		
	*Other central region district*	*1.7*	*1.06*	*(0.75–1.51)*	
	*Western region*	*0.79*	*0.84*	*(0.53–1.33)*	
	*Northern region*	*0.74*	*0.90*	*(0.50–1.62)*	
	*Eastern region*	*0.73*	*0.83*	*(0.50–1.37)*	
	*Outside Uganda*	*0.90*	*0.83*	*(0.28–2.48)*	
	*Household SES group (continuous, per unit)*	*0.91*	*0.96*	*(0.86–1.08)*	*0.5*
	*Date enrolled (continuous, per year)*	*1.11*	*1.05*	*(0.87–1.27)*	*0.64*
	*Family history of sickle cell*	*0.93*	*1.02*	*(0.58–1.78)*	*0..9*
Intermediate^2^	HIV positive	1.81	2.47	(1.73–3.54)	<0.0001
	Primigravida	2.04	1.40	(1.02–1.94)	0.04
	(Combined) Worm infections				<0.0001
	No *M perstans*, no hookworm	1.0	1.0		
	*M perstans*, no hookworm	2.85	2.39	(1.53–3.73)	
	No *M perstans*, hookworm	1.60	1.49	(1.08–2.06)	
	*M perstans* and hookworm	2.56	2.12	(1.46–3.08)	
	*S. mansoni infection*	*0.88*	*0.89*	*(0.62–1.27)*	*0.5*
	*Strongyloides infection*	*0.94*	*0.79*	*(0.52–1.19)*	*0.2*
	*Trichuris infection*	*1.04*	*0.96*	*(0.60–1.52)*	*0.9*
Proximate^3^	Taken any antimalarial in this pregnancy	0.56	0.63	(0.37–1.06)	0.09
	*Sprays bedroom/house*	*0.72*	*0.86*	*(0.60–1.22)*	*0.4*
	*Uses mosquito net*	*0.69*	*0.86*	*(0.66–1.13)*	*0.28*

1 Background risk factors adjusted for age, education and zone of residence.

2, 3 Intermediate and proximate risk factors adjusted for age, education, zone of residence, HIV status, gravidity and *Mansonella* and hookworm infection.

Variables that were omitted from the final models are shown in italics.

### Associations with HIV

Factors associated with HIV infection are shown in Table 4. Older women were at higher risk of having HIV, but education showed a strong protective effect. No association was seen with household socioeconomic status, tribe, or place of birth. Zone of residence, however, showed some evidence of an association, with a particularly high risk among women in the lakeside village of Kigungu. Using alcohol and reporting a history of past STIs were both associated with HIV. Increased gravidity was associated with HIV in the crude analysis, but not after adjusting for age. Widowed or divorced marital status showed good evidence of association with HIV, although partner risk factors were even better described by more specifically HIV-related questions on perceived and specific HIV risks. Exposure through fishing, either through the woman's or their partner's work, showed a crude but not an adjusted association with HIV. Bar work only showed a trend towards an effect. Having received a blood transfusion was a rare exposure and showed no statistical evidence of association with HIV.

**Table 4:** HIV.

Level	Risk Factor	Crude OR	Adjusted OR	(95% CI)	LR p-value
Background^1^	Age (continuous, per year)	1.07	1.07	(1.05–1.09)	<0.0001
	Education (continuous, per stage)	0.72	0.74	(0.62–0.88)	0.0008
	Zone of residence				0.07
	Entebbe	1.0	1.0		
	Kigungu	1.57	1.49	(1.03–2.16)	
	Abaita/Nkumba	0.78	0.78	(0.57–1.08)	
	Katabi, near main road	0.83	0.84	(0.54–1.30)	
	Katabi, away from main road	0.94	0.87	(0.55–1.38)	
	Unmapped				
	*Tribe*				*0.3*
	*Muganda*	*1.0*	*1.0*		
	*Munyankole*	*1.50*	*1.50*	*(1.00–2.22)*	
	*Mutoro*	*1.03*	*1.01*	*(0.53–1.92)*	
	*Musoga*	*0.63*	*0.64*	*(0.30–1.34)*	
	*Luo*	*0.86*	*0.72*	*(0.40–1.31)*	
	*Munyarwanda*	*1.12*	*0.97*	*(0.57–1.66)*	
	*Other*	*1.09*	*0.98*	*(0.72–1.35)*	
	*Place of birth*				*0.4*
	*Wakiso district*	*1.0*	*1.0*		
	*Other central region district*	*1.14*	*1.14*	*(0.81–1.61)*	
	*Western region*	*1.16*	*1.11*	*(0.73–1.69)*	
	*Northern region*	*1.12*	*0.94*	*(0.56–1.58)*	
	*Eastern region*	*0.75*	*0.72*	*(0.44–1.17)*	
	*Outside Uganda*	*0.89*	*0.79*	*(0.27–2.37)*	
	*Household SES group (continuous, per unit)*	*0.93*	*0.95*	*(0.86–1.06)*	*0.4*
	*Date enrolled (continuous, per year)*	*1.04*	*1.05*	*(0.88–1.26)*	*0.6*
Intermediate^2^	Marital Status				0.004
	Married	1.0	1.0		
	Single	0.99	1.26	(0.87–1.83)	
	Widowed	7.78	5.37	(1.82–15.8)	
	Divorced/separated	2.78	1.99	(1.06–3.73)	
Proximate^3^	Ever had an STD	1.79	1.45	(1.11–1.90)	0.007
	Uses alcohol	2.18	1.79	(1.37–2.34)	<0.0001
	HIV exposure				<0.0001
	No partner risk factors, no perceived risk	1.0	1.0		
	No partner risk factors, perceived risk	1.84	1.69	(1.28–2.23)	
	Partner risk factors, no perceived risk	2.48	1.72	(0.56–5.26)	
	Partner risk factors and perceived risk	25.3	18.4	(10.7–31.7)	
	*Primigravida*	*0.50*	*0.93*	*(0.64–1.36)*	*0.7*
	*Occupation exposure (woman or partner)*				
	*Fishing*	*1.31*	*1.00*	*(0.66–1.51)*	*1.0*
	*Bar work*	*1.65*	*1.42*	*(0.86–2.32)*	*0.2*

1, 2 Background and intermediate risk factors adjusted for age and education.

3 Proximate risk factors adjusted for age, education, history of sexually transmitted disease (STD), use of alcohol, partner risk factors and perceived risk.

Variables that were omitted from the final models are shown in italics.

### Associations with rare infections

Risk factors for *Ascaris*, *Trichostrongylus* and active syphilis have not been presented in detail because the low prevalence of these infections (<2.5% of women in this population) gave limited power to detect associations. However, the following was noted: education was protective for both *Ascaris* and syphilis (adjusted p<0.01); both *Ascaris* and syphilis were associated with location (adjusted p<0.01), with a particularly high prevalence in Kigungu; both *Ascaris* and *Trichostrongylus* reduced in prevalence over time (adjusted p = 0.002 for *Trichostrongylus*, 0.06 for *Ascaris*); and both *Ascaris* and *Trichostrongylus* were associated with not using tap water (adjusted p<0.05).

## Discussion

We have assessed risk factors associated with infections with helminths, *P. falciparum* and HIV among a sample of 2507 pregnant women attending Entebbe hospital who were enrolled into a randomized controlled trial over a two year period. We found that demographic, socioeconomic, geographic and behavioral risk factors all played a role, together with specific exposures for some particular outcomes, and that the prevalence of some infections changed over time. Our findings can be interpreted to guide public health interventions; they also identify potential confounders to be considered during further analysis of the effects of these infections in pregnancy on health outcomes. In particular, they add to prior work which focused specifically on the extent to which geography explained associations between helminth and malaria infections [26] by putting together a full picture of the risk factors affecting each infection.

This was a large study, enhanced by collection of geographical data, but the cross-sectional design of the analysis meant that we had to infer causality. Without a control group of non-pregnant women, we cannot assess which of our findings may have been pregnancy-specific, and some outcomes and exposures remained too rare to be assessed. Also, having examined many associations, there was potential to report some incorrect results through random error despite pre-censoring by considering likely causal connections. There was the potential for low sensitivity to detect intestinal helminth status (especially for *S. mansoni* and low intensity infections with other helminths) by Kato-Katz or culture, since this was based on examination of only part of a single stool sample [27],[28]. Similarly, microscopy is unable to diagnose *P. falciparum* malaria when this is sequestered in the placenta [29] and HIV serology would have missed women in the process of seroconversion. These limitations may have resulted in under-estimation of prevalence and of the strength of some associations, and thus the findings presented are likely to be conservative. Similarly, exclusion of women who were anaemic, or who had symptoms suggestive of helminth infections, may have resulted in exclusion of women with heavier infections, and weakened observed associations, but the number of participants excluded for these reasons was small and therefore unlikely to have any major impact on the findings.

A key finding regarding background risk factors was that age and education were associated with all high prevalence and some low prevalence infections, often with a strong association; younger or less educated women were at increased risk of infection with helminths or *P. falciparum*, and older or less educated women at risk of infection with HIV. This suggests that empowering women with education may have direct benefits for their health, that the needs of adolescent women who are out of school should be addressed particularly, and that older pregnant women are more likely to need antiretroviral therapy and interventions for prevention of mother-to-child HIV transmission. Only hookworm, *M. perstans* and *Strongyloides* showed an adjusted association with lower household socioeconomic status. However, the absence of an association for other infections should be interpreted with caution, since there was a limited variability in income within our population. A person's tribe and/or their place of birth, which might represent a number of other factors, but most of all past exposure to areas of different prevalence, showed an association with all helminths. Thus the association of *M. perstans* with origin in Central Uganda reflects the distribution of *M. perstans* as a transverse belt across Uganda and its absence from Northern regions where, instead, *Wuchereria bancrofti* is prevalent [30]. Similarly, schistosomiasis among women from the North and from Wakiso reflects the prevalence of the infection along the River Nile and its catchment, and around Lake Victoria [31]. On the other hand, associations with location of residence within the Entebbe peninsula emphasize the importance of current local environment. Together, these findings suggest the need to address both treatment of remotely acquired infections (especially considering that less than half of women reported ever having received prior worm treatment), and preventing local transmission within the Entebbe area. Over the course of enrolment there was a downward trend in hookworm, *Trichuris*, *Ascaris* and *Trichostrongylus* infection, suggestive of a population-level effect either of increased treatment of these infections in the community and among young children [32], or of interrupted transmission through improved hygiene and sanitation.

Regarding more direct risk factors for helminths, we found hygiene-related factors had a greater effect before adjusting for age, education, socioeconomic status and location, which suggests that this may have been part of the mode of action of these background variables. Alternatively; since several prior analyses have shown associations with toilet facilities (for which our population was quite homogenous) and other hygiene factors on which we did not have data such as type of garbage disposal [33] a mud floor versus a concrete or tiled floor [34], or handwashing [35], there may have been effects which we were not able to observe. History of anthelmintic treatment showed a strong effect, crude and adjusted, on hookworm, *M. perstans*, or *Strongyloides* infection, but not on *S. mansoni* or *Trichuris*. The effect on hookworm and the lack of an effect on *S. mansoni* are not surprising given the high proportion of women reporting use of benzimidazoles, the low proportion reporting praziquantel and the tendency of *Trichuris* to be resistant to single-dose treatment [36]. This prior treatment, affecting 9% of the study population, may weaken the associations observed between the affected helminths and key risk factors in the current analysis, and the observed effects of de-worming as the study proceeds. The association of a history of treatment with *M. perstans* is surprising, given prior reports that even long courses of benzimidazoles have limited efficacy in clearing this infection [37]; however, this and other treatment effects may at least partly reflect unmeasured underlying differences in women's behaviour.

The inverse association between HIV and hookworm infection, and between CD4 count and hookworm infection among HIV-positive women, is consistent with our own previous results and several other reports ([38]–[42]. As discussed elsewhere, this inverse association could result from a beneficial effect of hookworm in relation to HIV infection, but it is perhaps more likely that the association arises from an HIV-induced change in the immunological environment, or in the host intestine, which renders conditions unfavourable to hookworm infection [43],[44].

In relation to malaria, bed nets had a crude, but not an adjusted, effect; thus net use may have been part of the mode of action of age, education, and zone of residence. However, we had no data on insecticide treatment, net quality, or consistency of net use, and adjusting for zone of residence may have hidden population-level effects of varying bed net use. We saw only weak adjusted evidence for an effect of treatment, but the small no-treatment group gave limited power to see an association. The effect of primigravidity agreed with what is already well-known. The observed associations between helminths, malaria and the location of residence have been discussed in detail by Hillier et al. [26], who concluded that there was an association between *M. perstans* and *P. falciparum*, and a weaker association between hookworm and *P. falciparum*.

For HIV, the strong association with variables related to sexual history/behavior and STIs is no surprise, although some of the former may reflect biased reporting based on knowledge of HIV status. The absence of any suggestion of an effect of blood transfusion, while reassuring, may partly have been because this was a rare exposure. However, a particular point of interest was the high prevalence of both HIV and syphilis in the fishing village of Kigungu.

To summarize, as reported previously, our study population had a high prevalence (>10%) of hookworm, *S. mansoni*, *M. perstans*, *Strongyloides*, HIV and *P. falciparum* parasitemia. The health consequences of HIV and malaria for mother and baby are known to be negative and interventions to be effective [15],[16],[45], but the health consequences of helminth infections and their treatment, especially during pregnancy, are yet to be fully ascertained. Data from follow up in this study will inform policy in this area but definite recommendations for the management of helminths during pregnancy cannot be made at this time. There are, however, important public health messages which can be taken from this study and acted on immediately. First, education and interventions regarding both helminths and malaria are particularly needed for young women both in and out of school; second, the high prevalence of HIV and malaria highlights the need for enhanced effort in the prevention of mother-to-child transmission of HIV (PMTCT) and for interventions against malaria during pregnancy in this region; and last but not least, residents of fishing villages, represented in this study by Kigungu, stand to benefit particularly from syphilis, HIV and schistosomiasis interventions.

## Supporting Information

Table S1 *Mansonella perstans*
(0.08 MB DOC)

Table S2 *Strongyloides*
(0.10 MB DOC)

Table S3 *Trichuris trichiura*
(0.10 MB DOC)
