# Supplementary material for: Risk Factors for Helminth, Malaria, and HIV Infection in Pregnancy in Entebbe, Uganda
Source: PLoS Negl Trop Dis. 2009 Jun 30;3(6):e473. doi: 10.1371/journal.pntd.0000473 (PMC2696595; doi:10.1371/journal.pntd.0000473)
Supplement: Table S1 — Mansonella perstans (0.08 MB DOC) [file pntd.0000473.s001.doc]

***Table S1: Mansonella perstans***

| Level | Risk Factor | Crude OR | Adjusted OR | (95% CI) | LR p-value |
| --- | --- | --- | --- | --- | --- |
| Background^1^ | Age (continuous, per year) | 0.93 | 0.93 | (0.91-0.95) | <0.0001 |
|  | Education (continuous, per stage) | 0.84 | 0.82 | (0.70-0.97) | 0.02 |
|  | Tribe |  |  |  | <0.0001 |
|  | Muganda | 1.0 | 1.0 |  |  |
|  | Munyankole | 0.33 | 0.43 | (0.25-0.73) |  |
|  | Mutoro | 0.52 | 0.77 | (0.39-1.51) |  |
|  | Musoga | 1.56 | 3.83 | (2.04-7.19) |  |
|  | Luo | 0.12 | 0.60 | (0.19-1.86) |  |
|  | Munyarwanda | 0.91 | 0.76 | (0.49-1.16) |  |
|  | Other | 0.53 | 1.06 | (0.74-1.54) |  |
|  | Place of birth |  |  |  | <0.0001 |
|  | Wakiso district | 1.0 | 1.0 |  |  |
|  | Other central region district | 1.16 | 1.20 | (0.92-1.56) |  |
|  | Western region | 0.48 | 0.70 | (0.43-1.14) |  |
|  | Northern region | 0.11 | 0.12 | (0.04-0.33) |  |
|  | Eastern region | 0.73 | 0.44 | (0.26-0.75) |  |
|  | Outside Uganda | 0.57 | 0.52 | (0.20-1.33) |  |
|  | Household SES group (continuous, per unit) | 0.84 | 0.85 | (0.78-0.92) | 0.0002 |
|  | *Zone of residence* |  |  |  | *0.1* |
|  | *Entebbe* | *1.0* | *1.0* |  |  |
|  | *Kigungu* | *1.34* | *1.21* | *(0.86-1.71)* |  |
|  | *Abaita/Nkumba* | *1.03* | *0.99* | *(0.76-1.29)* |  |
|  | *Katabi, near main road* | *1.74* | *1.46* | *(1.05-2.02)* |  |
|  | *Katabi, away from main road* | *1.87* | *1.41* | *(0.99-2.01)* |  |
|  | *Unmapped* | *1.78* | *1.34* | *(0.67-2.66)* |  |
|  | *Date enrolled (continuous, per year)* | *1.05* | *1.00* | *(0.86-1.15)* | *1.0* |
| Intermediate^2^ | *HIV positive* | *1.06* | *1.25* | *(0.91-1.71)* | *0.2* |
|  | *Water source* |  |  |  | *0.2* |
|  | *Tap* | *1.0* | *1.0* |  |  |
|  | *Stand Pipe* | *1.25* | *1.12* | *(0.89-1.40)* |  |
|  | *Bore Hole* | *1.27* | *1.18* | *(0.78-1.79)* |  |
|  | *Well* | *0.89* | *0.87* | *(0.55-1.39)* |  |
|  | *Lake* | *0.83* | *0.60* | *(0.34-1.03)* |  |
|  | *Primigravida* | *1.32* | *0.93* | *(0.72-1.20)* | *0.6* |
| Proximate^3^ | Any prior anthelmintic treatment |  |  |  | 0.0002 |
|  | Never | 1.0 | 1.0 |  |  |
|  | Only prior to this pregnancy | 0.56 | 0.66 | (0.53-0.84) |  |
|  | During this pregnancy | 0.46 | 0.57 | (0.37-0.86) |  |
|  | *Own room* | *0.79* | *0.71* | *(0.41-1.24)* | *0.2* |
|  | *Ever swims/bathes in lake* | *1.04* | *0.92* | *(0.74-1.13)* | *0.4* |

^1, 2, 3^ Background and intermediate and proximate risk factors adjusted for age, education, tribe, place of birth, household socioeconomic status (SES)

Variables that were omitted from the final models are shown in italics
