# Supplementary material for: Risk Factors for Helminth, Malaria, and HIV Infection in Pregnancy in Entebbe, Uganda
Source: PLoS Negl Trop Dis. 2009 Jun 30;3(6):e473. doi: 10.1371/journal.pntd.0000473 (PMC2696595; doi:10.1371/journal.pntd.0000473)
Supplement: Table S2 — Strongyloides (0.10 MB DOC) [file pntd.0000473.s002.doc]

***Table S2: Strongyloides***

| Level | Risk Factor | Crude OR | Adjusted OR | (95% CI) | LR p-value |
| --- | --- | --- | --- | --- | --- |
| Background^1^ | Age (grouped) |  |  |  | 0.03 |
|  | 14-19 | 1.0 | 1.0 |  |  |
|  | 20-24 | 0.68 | 0.67 | (0.50-0.91) |  |
|  | 25-29 | 0.73 | 0.76 | (0.54-1.08) |  |
|  | 30-34 | 0.50 | 0.50 | (0.30-0.83) |  |
|  | 35+ | 0.81 | 0.75 | (0.40-1.41) |  |
|  | Education (continuous, per stage) | 0.74 | 0.78 | (0.65-0.95) | 0.01 |
|  | Place of birth |  |  |  | 0.02 |
|  | Wakiso district | 1.0 | 1.0 |  |  |
|  | Other central region district | 1.47 | 1.47 | (1.03-2.11) |  |
|  | Western region | 0.90 | 0.87 | (0.54-1.39) |  |
|  | Northern region | 1.15 | 1.16 | (0.67-1.99) |  |
|  | Eastern region | 1.54 | 1.67 | (1.07-2.61) |  |
|  | Outside Uganda | 1.07 | 0.96 | (0.32-2.85) |  |
|  | Household SES group (continuous, per unit) | 0.82 | 0.86 | (0.77-0.95) | 0.004 |
|  | *Tribe* |  |  |  | *0.3* |
|  | *Muganda* | *1.0* | *1.0* |  |  |
|  | *Munyankole* | *0.54* | *0.69* | *(0.36-1.33)* |  |
|  | *Mutoro* | *1.00* | *1.34* | *(0.59-3.04)* |  |
|  | *Musoga* | *1.41* | *1.47* | *(0.70-3.09)* |  |
|  | *Luo* | *0.97* | *1.27* | *(0.54-2.97)* |  |
|  | *Munyarwanda* | *1.73* | *1.52* | *(0.95-2.46)* |  |
|  | *Other* | *1.12* | *1.29* | *(0.82-2.04)* |  |
|  | *Zone of residence* |  |  |  | *0.3* |
|  | *Entebbe* | *1.0* | *1.0* |  |  |
|  | *Kigungu* | *1.48* | *1.41* | *(0.95-2.10)* |  |
|  | *Abaita/Nkumba* | *1.08* | *1.11* | *(0.81-1.52)* |  |
|  | *Katabi, near main road* | *1.09* | *1.22* | *(0.79-1.87)* |  |
|  | *Katabi, away from main road* | *1.61* | *1.48* | *(0.96-2.28)* |  |
|  | *Unmapped* | *0.71* | *0.69* | *(0.24-2.00)* |  |
|  | *Date enrolled (continuous, per year)* | *1.00* | *0.98* | *(0.82-1.17)* | *0.8* |
| Intermediate^2^ | *HIV positive* | *0.98* | *0.99* | *(0.68-1.46)* | *1.0* |
|  | *Water source* |  |  |  | *0.8* |
|  | *Tap* | *1.0* | *1.0* |  |  |
|  | *Stand Pipe* | *1.16* | *1.08* | *(0.81-1.43)* |  |
|  | *Bore Hole* | *1.46* | *1.29* | *(0.78-2.11)* |  |
|  | *Well* | *0.99* | *1.04* | *(0.60-1.81)* |  |
|  | *Lake* | *1.43* | *1.31* | *(0.74-2.33)* |  |
|  | *Primigravida* | *1.04* | *0.83* | *(0.59-1.17)* | *0.3* |
| Proximate^3^ | Any prior anthelmintic treatment |  |  |  | 0.007 |
|  | Never | 1.0 | 1.0 |  |  |
|  | Only prior to this pregnancy | 0.64 | 0.69 | (0.52-0.92) |  |
|  | During this pregnancy | 0.48 | 0.55 | (0.33-0.94) |  |
|  | Ever swims/bathes in lake | 1.54 | 1.50 | (1.15-1.96) | 0.003 |
|  | *Own room* | *0.96* | *0.84* | *(0.42-1.65)* | *0.6* |
|  | *Home toilet facilities* |  |  |  | *0.6* |
|  | *Pit latrine* | *1.0* | *1.0* |  |  |
|  | *Flush toilet* | *0.75* | *1.02* | *(0.59-1.74)* |  |
|  | *None* | *0.67* | *0.54* | *(0.16-1.81)* |  |
|  | *Ever walks in yard barefoot* | *1.28* | *1.10* | *(0.80-1.52)* | *0.6* |

^1, 2^ Background and intermediate risk factors adjusted for age, education, place of birth and household socioeconomic status (SES),

^3^  Proximate risk factors adjusted for age, education, place of birth, household socioeconomic status (SES), prior anthelmintic treatment and swimming in the lake.

Variables that were omitted from the final models are shown in italics
