# Supplementary material for: Risk Factors for Helminth, Malaria, and HIV Infection in Pregnancy in Entebbe, Uganda
Source: PLoS Negl Trop Dis. 2009 Jun 30;3(6):e473. doi: 10.1371/journal.pntd.0000473 (PMC2696595; doi:10.1371/journal.pntd.0000473)
Supplement: Table S3 — Trichuris trichiura (0.10 MB DOC) [file pntd.0000473.s003.doc]

***Table S3: Trichuris trichiura***

| Level | Risk Factor | Crude OR | Adjusted OR | (95% CI) | LR p-value |
| --- | --- | --- | --- | --- | --- |
| Background^1^ | Age (grouped) |  |  |  | <0.0001 |
|  | 14-19 | 1.0 | 1.0 |  |  |
|  | 20-24 | 0.50 | 0.51 | (0.36-0.71) |  |
|  | 25-29 | 0.40 | 0.42 | (0.28-0.63) |  |
|  | 30-34 | 0.48 | 0.51 | (0.30-0.88) |  |
|  | 35+ | 0.32 | 0.31 | (0.13-0.75) |  |
|  | Education (continuous, per stage) | 0.66 | 0.71 | (0.57-0.89) | 0.003 |
|  | Tribe |  |  |  | 0.008 |
|  | Muganda | 1.0 | 1.0 |  |  |
|  | Munyankole | 1.10 | 1.15 | (0.70-1.90) |  |
|  | Mutoro | 2.26 | 2.27 | (1.28-4.00) |  |
|  | Musoga | 0.75 | 0.74 | (0.33-1.65) |  |
|  | Luo | 0.48 | 0.42 | (0.18-1.00) |  |
|  | Munyarwanda | 1.94 | 1.58 | (0.95-2.66) |  |
|  | Other | 0.94 | 0.95 | (0.65-1.38) |  |
|  | Zone of residence |  |  |  | <0.0001 |
|  | Entebbe | 1.0 | 1.0 |  |  |
|  | Kigungu | 4.31 | 4.18 | (2.79-6.25) |  |
|  | Abaita/Nkumba | 1.58 | 1.62 | (1.12-2.37) |  |
|  | Katabi, near main road | 0.93 | 1.05 | (0.58-1.91) |  |
|  | Katabi, away from main road | 2.19 | 2.27 | (1.36-3.78) |  |
|  | Unmapped |  |  |  |  |
|  | Date enrolled (continuous, per year) | 0.82 | 0.78 | (0.63-0.96) | 0.02 |
|  | *Place of birth* |  |  |  | *0.5* |
|  | *Wakiso district* | *1.0* | *1.0* |  |  |
|  | *Other central region district* | *0.76* | *0.75* | *(0.51-1.08)* |  |
|  | *Western region* | *0.93* | *0.83* | *(0.45-1.54)* |  |
|  | *Northern region* | *0.40* | *0.50* | *(0.19-1.34)* |  |
|  | *Eastern region* | *0.52* | *0.57* | *(0.27-1.18)* |  |
|  | *Outside Uganda* | *1.15* | *1.00* | *(0.34-2.94)* |  |
|  | *Household SES group (continuous, per unit)* | *0.85* | *0.93* | *(0.82-1.05)* | *0.2* |
| Intermediate^2^ | *HIV positive* | *1.04* | *1.02* | *(0.66-1.59)* | *0.9* |
|  | *Water source* |  |  |  | *0.3* |
|  | *Tap* | *1.0* | *1.0* |  |  |
|  | *Stand Pipe* | *1.08* | *0.95* | *(0.68-1.34)* |  |
|  | *Bore Hole* | *1.55* | *1.31* | *(0.75-2.30))* |  |
|  | *Well* | *1.59* | *1.46* | *(0.82-2.59)* |  |
|  | *Lake* | *2.96* | *1.59* | *(0.89-2.84)* |  |
|  | *Primigravida* | *1.45* | *1.07* | *(0.74-1.56)* | *0.7* |
| Proximate^3^ | Ever swims/bathes in lake | 1.64 | 1.37 | (0.99-1.88) | 0.05 |
|  | *Any prior anthelmintic treatment* |  |  |  | *0.9* |
|  | *Never* | *1.0* | *1.0* |  |  |
|  | *Only prior to this pregnancy* | *0.82* | *0.92* | *(0.67-1.26)* |  |
|  | *During this pregnancy* | *0.65* | *0.92* | *(0.52-1.64)* |  |
|  | *Own room* | *0.73* | *0.61* | *(0.27-1.38)* | *0.2* |
|  | *Home toilet facilities* |  |  |  | *0.3* |
|  | *Pit latrine* | *1.0* | *1.0* |  |  |
|  | *Flush toilet* | *0.80* | *1.03* | *(0.56-1.92)* |  |
|  | *None* | *2.62* | *2.03* | *(0.84-4.89)* |  |
|  | *Ever walks in yard barefoot* | *1.46* | *1.29* | *(0.88-1.89)* | *0.2* |

^1, 2, 3^ Background, intermediate and proximate risk factors adjusted for age, education, tribe, zone of residence and date enrolled.

Variables that were omitted from the final models are shown in italics
